# Concomitant composite adrenal pheochromocytoma, multiple gastric stromal tumours and pseudohermaphrodism in a patient with von Recklinghausen's disease

**DOI:** 10.1186/1477-7800-3-11

**Published:** 2006-04-26

**Authors:** Dean Lisewski, Simon Ryan, Ee Mun Lim, Felicity Frost, Hieu Nguyen

**Affiliations:** 1 Department of Surgery, Sir Charles Gairdner Hospital, Nedlands, Western Australia; 2 The Western Australian Centre for Pathology & Medical Research (PathCentre), Nedlands, Western Australia

## Abstract

Although pheochromocytoma occurs in 1% of patients with von Recklinghausen's disease, composite tumors in this syndrome are much rarer, with isolated case reports in the literature. Most gastrointestinal stromal tumors (GISTs) are solitary and sporadic. Multiple GISTs however, are associated with clinical syndromes particularly von Recklinghausen's disease. We believe this is the first report of composite adrenal pheochromocytoma and multiple GISTs occurring in an 82 year old woman with neurofibromatosis type 1 (NF1), manifested by clitoral and subcutaneous neurofibromas, epilepsy and Lisch nodules. The extreme clitoromegaly raised concerns of pseudohermaphrodism at presentation.

## Introduction

Neurofibromatosis type 1 (NF1) is a heterogeneous, dominantly inherited disorder associated with a large number of different neoplasms, both benign and malignant. Pheochromocytoma occurs in 1% of patients with NF1, whereas composite tumors of the adrenal medulla are much rarer. By definition, a composite tumor consists of pheochromocytoma with neuroblastoma, ganglioneuroblastoma or ganglioneuroma. Both cell lines, the chromaffin cells and the symapathetic ganglion cells are of neural crest origin. Gastrointestinal stromal tumors (GISTs) are the most common mesenchymal neoplasms of the gastrointestinal tract and have also been described in association with NF1. GISTs are immunohistochemically positive for KIT tyrosine kinase receptor and this characteristic is also found in the interstitial cells of Cajal, the pacemaker cells regulating autonomic motor activity. It is postulated that GISTs originate from a multipotential precursor cells, which differentiate to an interstitial cell of Cajal phenotype. Neurofibromas of the clitoris have been reported infrequently. It represents a differential diagnosis that needs to be considered in pseudohermaphrodism.

NF1 has been associated, albeit rarely, with composite ganglioneuroma-ganglioneuroma of the adrenal medulla, multifocal gastric GISTs and neurofibroma of the clitoris, but we believe this is the first case report whereby all these disorders occur in the same patient with NF1.

## Case report

An 82-year old widow and mother of three with neurofibromatosis was hospitalized following a seizure due to non-compliance of anticonvulsant medication Her epilepsy had been previously well controlled on phenytoin 300 mg daily. Cranial computed tomography (CT) revealed an old left frontal cerebral infarct. The patient's other past history included mild aortic stenosis, fractured left neck of femur and iron deficiency anemia. Gastroscopy and colonoscopy were performed 3 years previously, when she was investigated for melena and no obvious cause found. Glibenclamide 5 mg tds had been commenced by her general practitioner for symptomatic glycosuria. She was on no other medication.

During this admission, the patient developed a gastrointestinal pseudo-obstruction, which was managed conservatively. It was during the investigation of her constipation and progressive abdominal distension that clitoromegaly was noted on examination and a 2.5 cm left adrenal mass found on abdominal CT.

The patient gave a history of clitoral enlargement from birth and recalls clitoral reduction surgery being offered and declined at 4-years of age. She developed numerous subcutaneous nodules at 16-years of age and cutaneous neurofibroma was confirmed on biopsy of the skin nodule at that time. There was a family history of neurofibromatosis, which afflicted both her maternal grandmother and great-grandmother, and the patient's own youngest daughter.

Given the association of neurofibromatosis and pheochromocytoma, the patient was questioned regarding paroxysmal symptoms of palpitations, headaches, sweats, flushing or visual disturbance, but these were absent. On examination she was normotensive with a postural drop from 135/60 mmHg to 110/60 mmHg. The pulse rate was 80 and regular and there was an audible, soft, aortic stenotic murmur. Widespread cutaneous neurofibromas and café-au-lait patches were noted. Clitoromegaly was present with the clitoris measuring 10 × 4 × 4 cm. The visual fields and fundi were normal although Lisch nodules were present on the left iris. The patient had bilateral sensineural hearing loss but no other neurological deficits were detected.

Biochemical investigations confirmed elevated 24-hour urinary catecholamine excretion 6 days post admission: adrenaline 232 nmol/day (reference interval <120 nmol/day), noradrenaline 465 nmol/day (ref. <600); and 24 hr urinary creatinine 4.6 mmol/day (ref. 5.0–16.0). A repeat 24 hr urinary catecholamine three days later also confirmed persisting elevation of urinary adrenaline of 149 nmol/day with urinary creatinine of 4.4 mmol/day. Urinary excretion of cortisol was 530 nmol/day (ref. <590). The patient was biochemically euthyroid with free thyroxine of 13 pmol/L (ref. 9–19) and TSH 3.15 mU/L (ref. 0.30–4.00). Prolactin was 380 mU/L (ref. <420) and she was normocalcemic, total plasma calcium corrected to albumin of 40 g/L was 2.47 mmol/L (ref. 2.25–2.60) and ionised calcium 1.18 mmol/L (ref. 1.12–1.32) adjusted to pH of 7.4. The serum sex hormone profile revealed testosterone 3.1 nmol/L (ref. <3.5), estradiol 110 pmol/L (ref. 40–130), free androgen index 2.3 (ref. 1–5), DHEA-sulphate <0.2 μmol/L (ref. 0.4–2.4), and androstenedione 4.4 nmol/L (ref. 1.6–9.3). Her serum 17-hydroxy progesterone was mildly elevated at 2.3 nmol/L (ref. 0.3–1.8) but this was felt to be of no clinical significance. Cytogenetic assessment of peripheral blood revealed a normal female karyotype.

Given the persistently elevated urinary adrenaline excretion, the CT finding of a 2.5 cm left adrenal tumor was likely to be functional and this was confirmed using ^123^I-MetaIodoBenzyl Guanidine (MIBG) scintigraphy. The patient was prepared for surgery with phenoxybenzamine 30 mg bd one week pre-operatively with propranolol 40 mg bd added the day prior to surgery. Laparoscopic left adrenalectomy was undertaken, but this was converted to an open adrenalectomy due to technical difficulties. During this procedure, approximately ten nodules measuring between 3 and 5 mm were noted on the serosal surface of the stomach, one of which was biopsied. Inspection of the rest of the intra-abdominal viscera was unremarkable. Core biopsies of the enlarged clitoris were taken. The post-operative course was uneventful and the patient made an excellent recovery. Repeat urinary catecholamine excretion prior to discharge was normal.

At two-month follow-up after her adrenalectomy, a clitorectomy was performed when a painful ulcer developed on the clitoris.

## Pathological findings

### Gastric serosal nodule

Macroscopically, the biopsy consisted of an irregular piece of firm pinkish tan tissue measuring 6 × 5 × 4 mm. Microscopically, there was a lobulated tumor composed of elongated uniform spindled cells separated by collagenous stroma. The nuclei were arranged in palisades and there was no cytological atypia and no mitoses were present. Small foci of tumor extended into the adjacent smooth muscle. Immunohistochemistry showed strong and diffuse staining for c-kit (CD117) and CD34. There was also focal staining for desmin and smooth muscle actin. There was no staining with S100. The findings were consistent with a small gastrointestinal stromal tumor (GIST).

### Clitoral biopsy and clitorectomy

The biopsy consisted of a core of soft, whitish grey tissue measuring 9-x-7x-1.5 mm and the entire tumor consisted of a skin covered polypoid mass measuring approximately 90 × 40 mm. There was a large kidney-shaped ulcerated area with well defined whitish margins measuring 43 × 30 mm. Microscopically there were small circumscribed nests of neural type structures reminiscent of sensory Meissnerian corpuscles separated by slightly myxoid stroma containing numerous mast cells. Focally, there was a diffuse architectural pattern of ill-defined fascicles of cytologically uniform spindle cells with abundant fibrillary matrix. Immunohistochemistry showed S100 positivity. Histological diagnosis was a clitoral neurofibroma.

### Left adrenal tumour

The left adrenal gland measured 48 × 25 × 50 mm and weighed 11 grams. Bulging above the surface was a nodule measuring 20 × 20 × 13 mm, which was variegated grey-yellow-orange on the cut surface. A 6 mm greyish nodule was found within the adjacent fatty tissue. Microscopically, the majority of the tumour (70%) showed typical features of a pheochromocytoma, with discrete nests of tumour cells separated by delicate fibro-vascular septa. These cells were of large with conspicuous granular cytoplasm and showed pleomorphism, many cells having distinct nucleoli. Mitotic figures were not seen and there was no necrosis. The cells extended through the capsule focally into the peri-adrenal fat. No vascular invasion was seen. In a few areas the tumour cells were more widely separated by thick bands of collagen. The second component of the tumour (30%) showed features of ganglioneuroma, with large mature ganglion-type cells with large nuclei, central eosinophilic nucleoli and abundant cytoplasm embedded within a fibrillary neural meshwork containing cytologically uniform spindle-shaped cells. No primitive neuroblastic elements were seen. The two tumour components were well demarcated in some areas, but focally appeared admixed.

Immunohistochemistry showed no staining of the tumour elements with the proliferation marker Ki-67 or p53 protein. Positive staining for S100 protein was identified in the sustentacular cells within normal adrenal medulla and in the phechromocytoma, as well as in the ganglion cells of the ganglioneuroma. The adrenal tumour was therefore a mixed pheochromocytoma/ganglioneuroma or composite pheochromocytoma.

The nodule noted adjacent to the main adrenal swelling consisted of an enlarged ganglion including expanded and distorted nerve fibres. The appearances suggested focal involvement by neurofibroma.

## Discussion

Neurofibromatosis type 1 (NF1) is one of the most common dominantly inherited disorders, with a birth incidence of 1 in 3000 [1]. The classic description given by the pathologist von Recklinghausen more than a century ago corresponds well with the most common clinical picture [2]. The National Institutes of Health Consensus Statement [3] outlined seven diagnostic criteria for NF1 and two or more criteria need to be present in order to make a diagnosis. These criteria include: 6 or more café-au-lait macules (size >5 mm in pre-pubertal patients and size >15 mm in post-pubertal patients); 2 or more neurofibromas or 1 or more plexiform neurofibromas; axillary or inguinal freckling; 2 or more Lisch nodules; optic gliomas; specific osseous dysplastic lesions; or a first-degree relative with NF1 diagnosed with the above criteria.

Expression of NF1 is highly variable. Patients with this disorder have an increased incidence of both benign and malignant tumours. This was shown in an investigation of 70 adult NF1 patients in Sweden [1]. Malignant tumours were reported four times as often in the NF1 group than in the general population. Of the NF1 group, 24% had developed malignant tumours (16% carcinoma, 7% sarcoma, 1% melanoma) and 17% had developed benign tumours (7% GIST, 6% pheochromocytoma, 3% adenoma and 1% meningioma). Pheochromocytomas (Pheo) are well recognised in the setting of NF1 and said to be 10 times more common than the general population [4]. NF1 is found in 5–25% of patients with Pheo [4], whereas the overall incidence of Pheo in NF1 is 1% [5].

Composite adrenal medullary tumours, on the other hand, are very rare. In an analysis from the National Cancer Institute of 120 adrenal and extra-adrenal Pheos, only four had a composite phenotype, making an overall incidence of 3% [7]. A review by Moore and Biggs [8] found only 13 cases of composite pheochromocytoma-ganglioneuroma (pheo-GN) reported in the literature. Of these, only three reports found composite Pheo-GN occurring in the setting of NF1 [6,9,11]. Our case report represents the fourth such reported occurrence. In addition, an adrenal ganglioneuroma and a contralateral Pheo in a patient with NF1 has been reported [10].

Bolande [12] was the first to propose the concept of neurocristopathies or tumours originating from neural crest-derived cells. These tumours of neural crest origin are composed of various combinations of adrenomedullary or extra-adrenal chromaffin cells, neuroblasts or sympathetic ganglion cells of varying stages of differentiation, either benign or malignant. Pheo, paraganglioma, GN, neuroblastoma and neurofibroma all represent such neurocristopathies.

Sympathogonia derived from certain neural crest cells differentiate into neuroblasts and pheochromoblasts. These neural crest-derived cells migrate ventrally and differentiate to give rise to the cellular components of the sympathetic ganglia and the adrenal medulla. Any migration disturbance or maldevelopment of the neural crest might result in the development of composite tumours. As these cell populations have similar derivation one might expect composite adrenal medullary tumours to be more common than what is apparent from the literature and this may represent under-reporting or under-recognition of this condition.

NF1 affects the gastrointestinal tract in 10–25% of cases [15] and it does so in four principle forms: stromal tumours, neuronal hyperplasia, ganglioneuromatosis and endocrine tumours of the duodenum and periampullary regions. Acute or recurrent gastrointestinal haemorrhage may occur in up to 40% of these patients [16]. Gastrointestinal stromal tumours (GISTs) are mesenchymal tumours of the gastrointestinal tract, which do not have any of the identifying features indicating an origin from a specific connective tissue cell. They differ from smooth muscle tumours both immunophenotypically and molecularly. Recently ultrastructural similarities have been found between GIST cells and the interstitial cell of Cajal (a gastrointestinal pacemaker cell) [13] and furthermore there are immunohistochemical similarities with both expressing KIT tyrosine kinase receptor. It has been suggested that GISTs may originate from stem cells that differentiate towards a pacemaker cell and smooth muscle cell. GISTs may express a number of different phenotypes, which has resulted in a multitude of acronyms used to describe them [14]. Myoid cell types have been called smooth muscle GISTs (smGIST), neural cell types called gastrointestinal autonomic nerve tumours (GANT), Cajal cell types called gastrointestinal pacemaker cell tumours (GIPACT) and mixed cell types (mixed GIST). The biological behaviour of GISTs has been difficult to predict. Multivariate analysis of survival data in 1004 GISTs has shown tumour size and location, mitotic rate and age to be important prognostic factors [17]. However, no method or combination of methods has proven to be reliable in determining malignancy in all cases.

Genitourinary neurofibromas are rare and clitoral involvement in NF1 has been reported infrequently. However, when it occurs, clitoromegaly is often the presenting sign. In many cases it is congenital. In a study of 236 families with NF1, four patients had clitoral involvement and in three, involvement was limited to the clitoris [18]. A review of the literature by Sulphen [18] found 26 patients with NF1 and clitoral involvement.

This case report illustrates the heterogeneity of NF1 with the concomitant presentation of three rare manifestations of this fascinating disorder, namely composite pheo-GN of the adrenal medulla, multiple gastric GIST and neurofibroma of the clitoris. To our knowledge this is the first time such a case has been reported in the literature.

**Figure 1:**
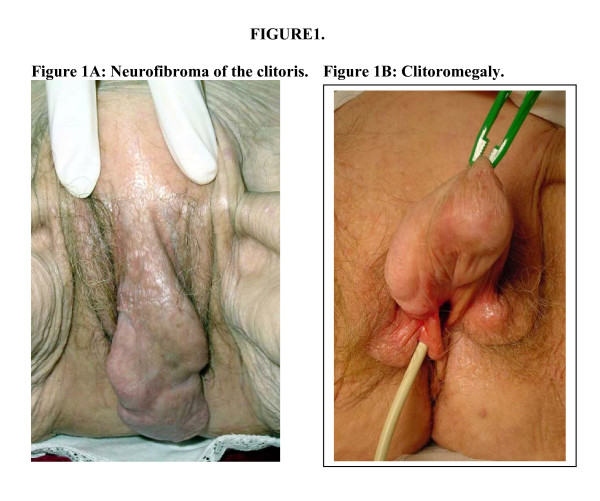
A: Neurofibroma of the clitoris. B: Clitoromegaly.

**Figure 2:**
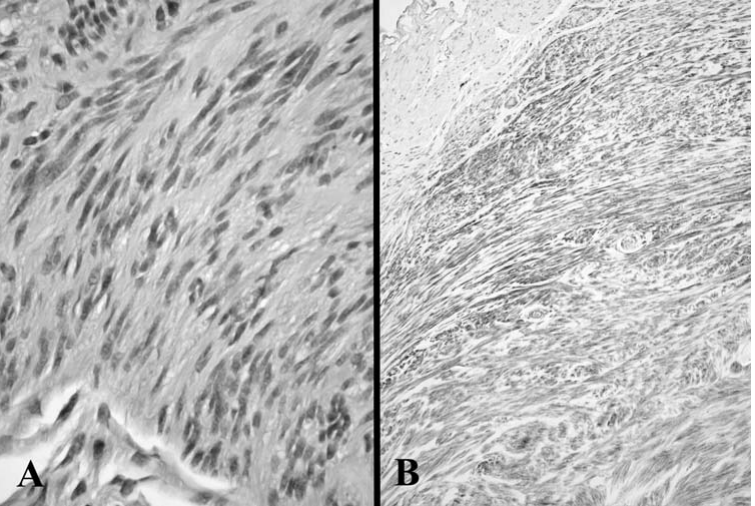
Gastric gastrointestinal stromal tumour (GIST) showing fascicles of uniform spindle cells (A, H&E original magnification × 250) and with c-kit staining of tumour cells compared to absent staining in adjacent smooth muscle at upper left (B, c-kit original magnification ×100).

**Figure 3:**
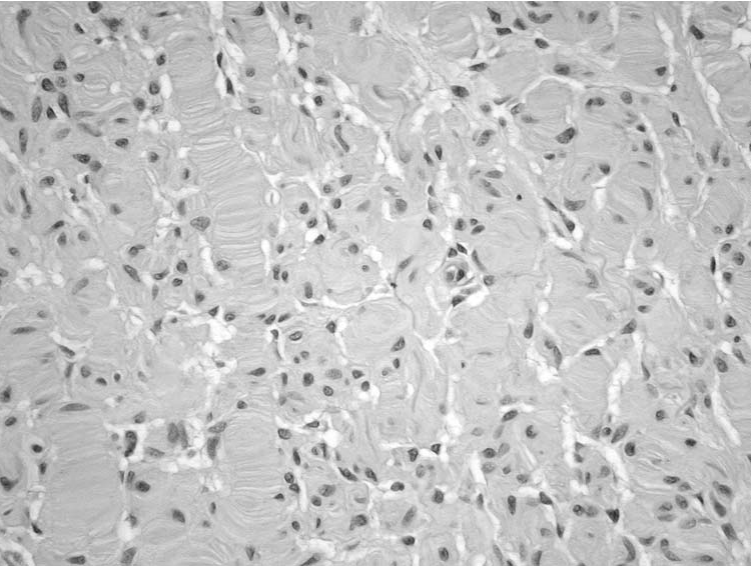
Neurofibroma of clitoris showing a growth pattern resembling Meissner bodies (H&E, original magnification × 250).

**Figure 4:**
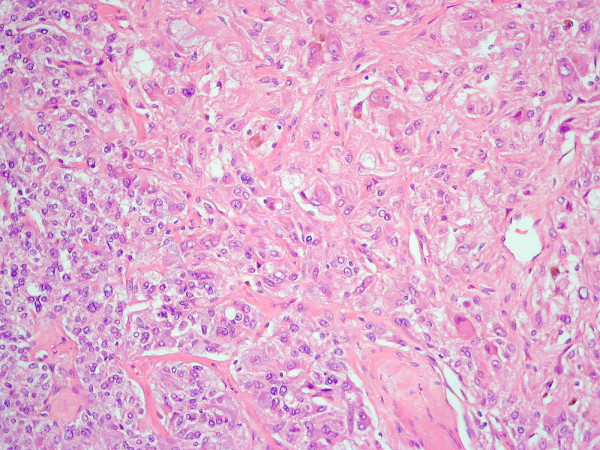
Composite adrenal tumour with phaeochromocytoma (left lower) merging with ganglioneuroma (upper right) (H&E, original magnification ×250).
